# Activation of Dopamine 4 Receptor Subtype Enhances Gamma Oscillations in Hippocampal Slices of Aged Mice

**DOI:** 10.3389/fnagi.2022.838803

**Published:** 2022-03-16

**Authors:** Yuan Wang, Yi-Kai Jin, Tie-Cheng Guo, Zhen-Rong Li, Bing-Yan Feng, Jin-Hong Han, Martin Vreugdenhil, Cheng-Biao Lu

**Affiliations:** ^1^Henan International Key Laboratory for Non-invasive Neuromodulation, Department of Physiology and Pathology, Xinxiang Medical University, Xinxiang, China; ^2^Department of Rehabilitation Medicine, Tongji Medical College, Tongji Hospital, Huazhong University of Science and Technology, Wuhan, China; ^3^School of Public Health, Xinxiang Medical University, Xinxiang, China; ^4^Department of Health Sciences, Birmingham City University, Birmingham, United Kingdom

**Keywords:** dopamine, γ oscillation, hippocampus, dopamine receptor, ageing

## Abstract

**Aim:**

Neural network oscillation at gamma frequency band (γ oscillation, 30–80 Hz) is synchronized synaptic potentials important for higher brain processes and altered in normal aging. Recent studies indicate that activation of dopamine 4 receptor (DR4) enhanced hippocampal γ oscillation of young mice and fully recovered the impaired hippocampal synaptic plasticity of aged mice, we determined whether this receptor is involved in aging-related modulation of hippocampal γ oscillation.

**Methods:**

We recorded γ oscillations in the hippocampal CA3 region from young and aged C57bl6 mice and investigated the effects of dopamine and the selective dopamine receptor (DR) agonists on γ oscillation.

**Results:**

We first found that γ oscillation power (γ power) was reduced in aged mice compared to young mice, which was restored by exogenous application of dopamine (DA). Second, the selective agonists for different D1- and D2-type dopamine receptors increased γ power in young mice but had little or small effect in aged mice. Third, the D4 receptor (D4R) agonist PD168077 caused a large increase of γ power in aged mice but a small increase in young mice, and its effect is blocked by the highly specific D4R antagonist L-745,870 or largely reduced by a NMDAR antagonist. Fourth, D3R agonist had no effect on γ power of either young or aged mice.

**Conclusion:**

This study reveals DR subtype-mediated hippocampal γ oscillations is aging-related and DR4 activation restores the impaired γ oscillations in aged brain, and suggests that D4R is the potential target for the improvement of cognitive deficits related to the aging and aging-related diseases.

## Introduction

Neural oscillations in the gamma frequency band (γ oscillations, 30–120 Hz) represent the synchronized neuronal activity of populations of neurons that, by millisecond precision of synaptic activity, aids effective communication (Chrobak and Buzsaki, 1998), which plays a crucial role in information processing in cortical networks (Uhlhaas et al., 2011). Parvalbumin (PV)-expressing fast-spiking interneurons that control the firing of principal cells, and somatostatin-expressing interneurons that control their activity are essential in generating slow (< 60 Hz) γ oscillations (Antonoudiou et al., 2020).

γ oscillations are closely associated with cognitive function (Mann et al., 2005; Fuchs et al., 2007; Cardin et al., 2009; Sohal et al., 2009). γ oscillations are modulated by neurotransmitters such as dopamine (DA), a catecholamine neurotransmitter that mediates multiple brain functions, including cognition, emotion and motor coordination (Florán et al., 2015). Recent studies in rodents demonstrate that dopaminergic modulation of hippocampal γ oscillations is mediated by dopamine receptors (DR)1 and DR2 (Weiss et al., 2003; Xie et al., 2021), DR3 (Schulz et al., 2012), as well as DR4 (Andersson et al., 2012a,b).

Normal aging is associated with a gradual decline of some cognitive functions, especially those dependent on hippocampal function (Driscoll et al., 2003) and is associated with a reduction in γ oscillation power in both rodents (Vreugdenhil and Toescu, 2005; Lu et al., 2012) and humans (Murty et al., 2020). Studies about the DA levels and DA synthesis capacity are controversial, some reported DA synthesis capacity are relatively preserved in aging (Karrer et al., 2017; Von Linstow et al., 2017), but Norrara et al., 2018 reported a significant decrease in the tyrosine hydroxylase immunoreactivity across nucleus accumbens in aged animals (Norrara et al., 2018). The expression of D1-like dopamine receptors is reduced in the hippocampus of aged rodents (Guo et al., 2017; Zhang et al., 2021) and humans (Karrer et al., 2017), whereas the expression of D2-like receptors is less or not age-dependent (Dang et al., 2017; Karrer et al., 2017; Seaman et al., 2019). Interestingly, the expression of DR4, a major D2-like receptor, prominently expressed in PV-expressing interneurons (Andersson et al., 2012b), is preserved in the aged mouse hippocampus (Guo et al., 2017). Given the γ oscillation-enhancing effect of DA, it is well possible that the age-related changes in DR expression contribute to the age-related reduction in γ oscillation. However, it is not known how normal aging affects the DA-mediated modulation of hippocampal γ oscillations. Given the enhancing effect of DR4 activation on gamma oscillations (Andersson et al., 2012a,b), the preserved expression of DR4 in aging and the full restoration of the impaired hippocampal LTP by the DR4 agonist PD168077 in aged mice (Guo et al., 2017), it is possible that DR4 activation might rescue the aging-impaired γ oscillations and related cognitive functions.

Hippocampal CA3 is a region enriched in recurrent collateral connections, which favors generation of synchronized network oscillation (Sun et al., 2017) and the circuitry responsible for slow γ oscillations is preserved in hippocampal slices, where slow γ oscillations can be elicited in CA3 area by application of nanomolar concentrations of kainate (Hormuzdi et al., 2001) or the cholinergic receptor agonist, carbachol (CCH) (Buhl et al., 1998; Gulyás et al., 2010; Stoiljkovic et al., 2015). These pharmacologically-induced γ oscillations are long-lasting and well-suitable for the mechanistic study of slow γ oscillations and their modulation by drugs that affect cognition (Buhl et al., 1998; Gulyás et al., 2010; Wang et al., 2017). In this study, we investigated the age-dependent modulation of different dopamine receptor subtypes on CCH-induced γ oscillations in hippocampal slices and establish that DR4 activation can rescue the aging-related decline in hippocampal γ oscillations.

## Materials and Methods

### Animals

All animal experiments followed the “Principles of laboratory animal care” (NIH publication No. 86−23, revised 1985) and the ARRIVE guidelines for the design, analysis, and reporting of scientific research as well as the guidelines and regulations of the Ethics Committees at the Xinxiang Medical University for the Care and Use of Laboratory Animals. The efforts were made to minimize animal suffering and the numbers of animals used. C57bl/J6 mice were purchased from Beijing Weitong Lihua Experimental Animal Co. and grouped as “young” (2–3 month old males) or “aged” (22–28 months old).

### Slice Preparation

Animals were anesthetized by intraperitoneal injection of Sagatal (sodium pentobarbitone, 100 mg/kg, Rhône Mérieux Ltd., Harlow, United Kingdom). When pedal reflexes were abolished, the animals were perfused intracardially with chilled (5°C) oxygenated artificial cerebrospinal fluid (ACSF), in which the sodium chloride had been replaced by iso-osmotic sucrose. This sucrose-ACSF contained (in mM): 225 sucrose, 3 KCl, 1.25 NaH_2_PO_4_, 24 NaHCO_3_, 6 MgSO_4_, 0.5 CaCl_2_, and 10 glucose (pH: 7.4). Horizontal slices (350 μm) of the mouse brain were cut in chilled sucrose-ACSF, using a Leica VT1000S vibratome (Leica Microsystems, Milton Keynes, United Kingdom). Slices containing the ventral hippocampus were stored at room temperature at the interface between recording ACSF and humidified carbogen (95% O_2_–5% CO_2_), until transferred to the Haas-type recording chamber. The recording ACSF contained (in mM): 126 NaCl, 3 KCl, 1.25 NaH_2_PO_4_, 24 NaHCO_3_, 2 MgSO_4_, 2 CaCl_2_, and 10 Glucose (pH: 7.4).

### Electrophysiological Recording, Data Acquisition, and Analysis

In the recording chamber, hippocampal slices (ventral hippocampus) were maintained at a temperature of 32°C, at the interface between ACSF (3 ml/min) and warm humidified carbogen (300 ml/min) and were allowed to equilibrate in this medium for 1 h prior to recording. With the help of a Leica MZ8 stereomicroscope at 20 x magnification, borosilicate glass microelectrodes, containing recording ACSF (resistance 2–5 MΩ), were placed at the stratum pyramidale/stratum lucidum border of area CA3c. Extracellular field potentials were amplified by Neurolog NL106 AC/DC amplifiers (Digitimer Ltd., Welwyn Garden City, United Kingdom) and band-pass filtered between 0.5 Hz and 2 kHz, using Neurolog NL125 filters (Digitimer). Electromagnetic interference from the mains supply was on-line eliminated from the recordings, with the use of Humbug 50 Hz noise eliminators (Digitimer Ltd.). The recordings were then continuously digitized at a sample rate of 2 kHz, using a CED 1401 plus ADC board (Cambridge Electronic Design, Cambridge, United Kingdom). Data were analyzed off-line, using Spike-2 software (Cambridge Electronic Design). Power spectra were generated to provide a quantitative measure of the frequency components. Power spectra were constructed for 60 s epochs, using a fast Fourier transform algorithm with a 2048 FFT size and a Hanning window. The integrated power between 20 and 60 Hz was used to quantify the power of the γ oscillations (γ power) at 32°C.

### Drugs

The DR1 agonist SKF-81297 (1 μM), the D2-like receptor agonist quinpirole (10 μM), the selective DR3 agonist pramipexole (10 μM), the DR4 agonist PD-168077 (200 nM), the selective DR4 antagonist L-745870 (500 nM), the dopamine (200 μM), the n-methy-d-aspartate (NMDA) receptor antagonist D-AP5 (50 μM) and CCH (5 μM) were purchased from Tocris Bioscience. Stock solutions, at thousand times the final concentration, were made up in water or in DMSO, and stored in individual aliquots at 20°C. Final solutions were prepared freshly on the day of the experiment. Drugs were applied to the recording ACSF 15 min after steady-state γ power was reached. The dead volume and bath volume was kept such that final concentration was reached in the bath in about 5 min.

### Statistical Methods

All statistical tests were performed using SigmaStat software (Sysstat software, San Jose, CA, United States). Where data sets were different from a normal distribution, results are expressed as median (interquartile range) and between-group differences were tested with a Mann-Whitney *U*-tests. Where data sets were not different from a normal distribution, results are expressed as mean ± standard error of the mean, between-group comparisons were made with a Student’s *t*-test and within-group comparisons were made with a paired Student’s *t*-test or a repeated measures ANOVA. Effects were considered statistically significant if *P* < 0.05.

## Results

### Cholinergic Receptor Agonist, Carbachol-Induced γ-Oscillations Are Reduced in Hippocampal Slices of Aged Mice

**FIGURE 1:**
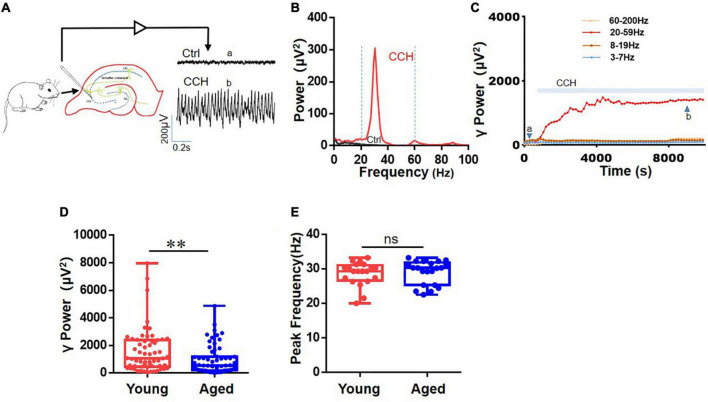
Effects of age on CCh-induced hippocampal γ oscillations. **(A)** Diagram shows γ oscillations was recorded in the CA3 region of the hippocampus from a young mouse. **(B)** Example of power spectrum of γ oscillations from the same young mouse. Two vertical dashed line (blue) showing the powers of the 20–60 Hz events. **(C)** Typical example of the time course of CCH-induced γ power in hippocampal CA3 area of young mouse. **(D)** Box and whisker plots of hippocampal γ power (summated power in 20–60 Hz range) in young and aged mice (young vs. aged γ power, ^**^P = 0.010). **(E)** Box and whisker plots of peak frequency of hippocampal oscillations from young and aged mice. Data are expressed as median and upper/lower quartile. ns: not significant.

Persistent oscillations were induced by CCH (5 μM) in area CA3 of hippocampal slices (example in Figure 1A). The power spectra for these recordings show a clear peak in the slow gamma range at 32°C (Figure 1B). The γ power (summated power in 20–60 Hz range) increased and took 40–120 min to reach a steady-state (< 5% change over 15 min), after which γ power was stable for several hours (Figure 1C), which allows assessing the effect of drugs on γ power and peak frequency. There was no difference in the time to reach steady-state γ power between the age groups. The γ power in 60 slices from 19 aged mice [552 (194, 1,189) μV^2^] was weaker than that in 59 slices from 19 young mice [1,069 (474, 2,384) μV^2^, *U* = 1,288, *P* = 0.010, Figure 1D]. There was no difference between the peak frequency of the γ oscillations in slices from young mice [29.3 (26.4, 30.6) Hz, *n* = 21 slices from 11 mice] and that in slices from aged mice [30.2 (25.3, 31.5) Hz, *n* = 22 slices from 11 mice, *U* = 246.5, *P* = 0.715, Figure 1E].

### The Effect of Dopamine on γ-Oscillations in Hippocampal Slices of Aged Mice

**FIGURE 2:**
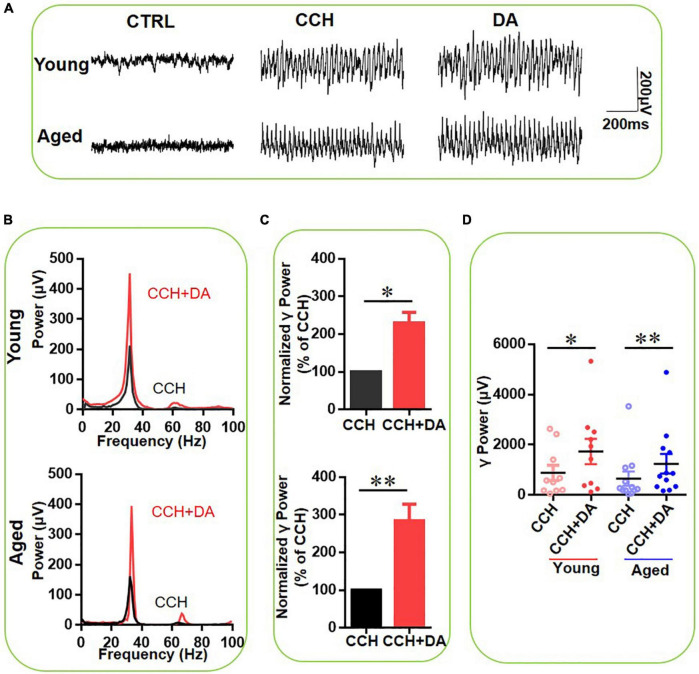
The effect of DA on CCH-induced γ oscillations in the young and aged mice. **(A)** Example traces of filed potentials recorded in hippocampal CA3 for CCH-induced γ oscillations in the presence of DA (200 μM) from a young and aged mouse. **(B)** Power spectra of the oscillatory activity in CCH alone (black line) and DA application (red line) for the slices shown in **(A)**. **(C)** The γ power normalized to the CCH only baseline value for CCH only application (CCH control) and for DA application. **(D)** Scatter plots of hippocampal γ power (summated power in 20–60 Hz range) in young and aged mice. The red circles represent CCH-induced γ power before (empty) and after (full) application of DA from the young mice. The blue circle represents the CCH-induced γ power before (empty) and after (full) application of DA from the aged mice [vs. CCH control, *t*_(9)_ = –2.896, *P = 0.018 for young mice; *t*_(11)_ = –3.877, ^**^P = 0.002 for aged mice]. Data are expressed as mean ± SEM. ns: not significant.

To study the age-dependent effect of DA on γ oscillations, DA (200 μM) was applied 15 min after CCH-induced γ power reached a steady state. The γ oscillations between 15 and 20 min after DA application were compared with those in the 5 min before DA application (baseline). Figure 2A gives an example of the effect of DA on the γ oscillation strength in a typical slice from a young mouse (top panels) and an aged mouse (bottom panels). The power spectrum of the oscillation in the slice from the young mouse (Figure 2B, top panel) and of that from the aged mouse (Figure 2B, bottom panel) show no change in peak frequency. DA increased γ power by 128 ± 26% from baseline [*t*_(9)_ = –2.896, *P* = 0.018] in ten slices from four young mice and by 185 ± 43% [*t*_(11)_ = –3.877, *P* = 0.002] in 12 slices from four aged mice (Figures 2C,D). In the presence of DA there was no significant difference in γ power between slices of young and aged mice [*t*_(21)_ = –0.794, *P* = 0.437]. DA had no effect on peak frequency of γ oscillation in either young [CCH 29.6 ± 0.6 Hz vs. CCH + DA 29.4 ± 0.7 Hz, *t*_(9)_ = 0.563, *P* = 0.587] or aged mice [CCH 28.9 ± 1.0 Hz vs. CCH + DA 28.7 ± 0.9 Hz, *t*_(10)_ = 0.473, *P* = 0.646]. These results indicate that the reduced γ-power in slices from aged mice can be restored by exogenous DA.

### Dopamine Receptors 1 Activation Increases γ Oscillations in Slices From Young Mice, but Not in Slices From Aged Mice

**FIGURE 3:**
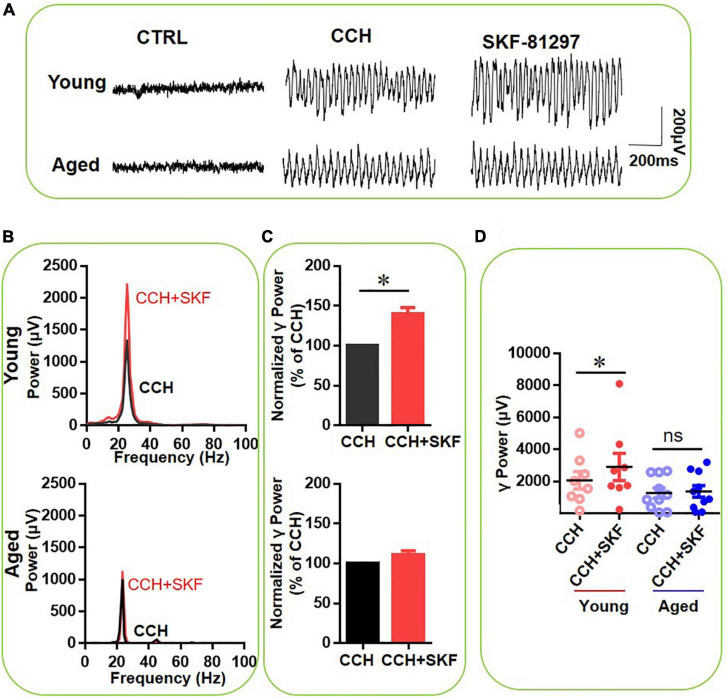
The influence of SKF-81297 on CCH-induced γ oscillations in the young and aged mice. **(A)** Example of γ oscillations recorded in area CA3, induced by CCH (5 μM) and after addition of SKF-81297 (1 μM). **(B)** Power spectra of the oscillatory activity in CCH alone (black line) and after SKF-81297 application (red line) for the slice in **(A)**. **(C)** The γ power normalized to the CCH only baseline value for CCH only application (control) and for SKF-81297 application. **(D)** Scatter plots of hippocampal γ power (summated power in 20–60 Hz range) in young and aged mice. The red circles represent CCH-induced γ power before (empty) and after (full) application of SKF-81297 from the young mice. The blue circle represents the CCH-induced γ power before (empty) and after (full) application of SKF-81297 from the aged mice [vs. CCH control, *t*_(7)_ = –2.953, *P = 0.021 for young mice]. Data are expressed as mean ± SEM. ns: not significant.

To investigate the contribution of DR1 activation to the effect of DA on γ oscillations, the selective DR1 agonist SKF-81297 (1 μM) was applied. Figure 3A gives an example of the effect of DA on the γ oscillation strength in a typical slice from a young mouse (top panels) and an aged mouse (bottom panels). The power spectrum of the oscillation in the slice from the young mouse (Figure 3B, top panel) and of that from the aged mouse (Figure 3B, bottom panel) show no change in peak frequency. SKF-81297 increased γ power of eight slices from three young mice by 40 ± 8% of baseline [*t*_(7)_ = –2.953, *P* = 0.021], but had no effect on γ power in 10 slices from of four aged mice [111 ± 5% of baseline power, *t*_(9)_ = –1.578, *P* = 0.149, Figures 3C,D]. There was a significant difference in SKF-81297-induced changes of hippocampal γ power between young and aged mice [*t*_(16)_ = 3.198, *P* = 0.006, Figures 3C,D]. These results indicate that DR1 activation contributes to DA-mediated modulation of hippocampal γ oscillations in young mice, but not in aged mice.

### D2-Like Receptor Activation Increases γ Oscillations More in Slices From Young Mice Than in Slices From Aged Mice

**FIGURE 4:**
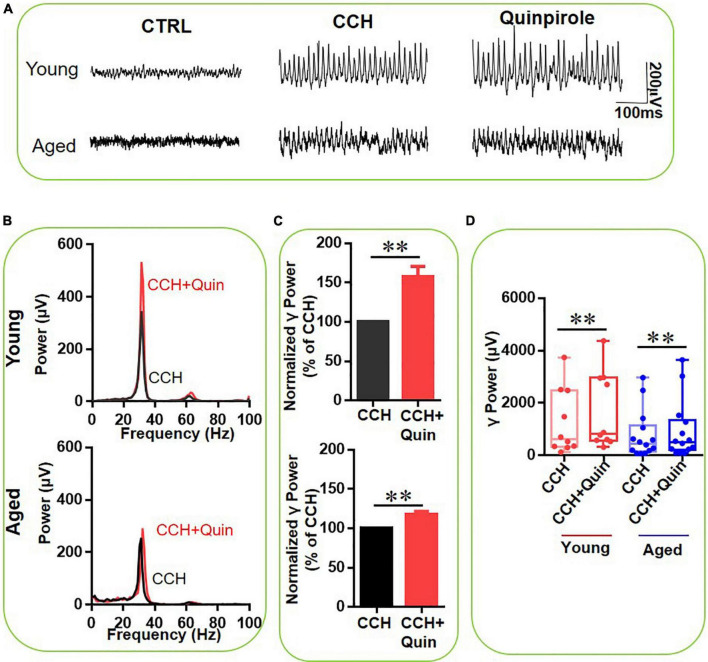
The influence of Quinpirole on CCH-induced γ oscillations in the young and aged mice. **(A)** Example of γ oscillations recorded in area CA3, induced by CCH (5 μM) and after addition of quinpirole (10 μM). **(B)** Power spectra of the oscillatory activity in CCH alone (black line) and after quinpirole application (red line) for the slice in **(A)**. **(C)** The γ power normalized to the CCH only baseline value for CCH only application (control) and for Quinpirole application. **(D)** Box and whisker plots of hippocampal γ power (summated power in 20–60 Hz range) in young and aged mice. The red circles represent CCH-induced γ power before (empty) and after (full) application of Quinpirole from the young mice. The blue circle represents the CCH-induced γ power before (empty) and after (full) application of Quinpirole from the aged mice (vs. CCH control, *Z* = 2.666, ^**^P = 0.005 for young mice; *Z* = 3.233, ^**^P = 0.002 for aged mice). Data are expressed as median and upper/lower quartile.

We then investigated the effect of the role of D2-like receptor activation in the DA-mediated increase of γ oscillations, by using the D2-like receptor agonist quinpirole (10 μM), which has a relative high affinity for DR2, but also binds to DR3 and DR4 (Surmeier et al., 1996). Figure 4A gives an example of the effect of quinpirole on the γ oscillation strength in a typical slice from a young mouse (top panels) and an aged mouse (bottom panels). The power spectrum of the oscillation in the slice from the young mouse (Figure 4B, top panel) and of that from the aged mouse (Figure 4B, bottom panel) show no change in peak frequency. Quinpirole increased hippocampal γ power by 57 ± 15% from baseline in 9 slices from 3 young mice (*Z-Statistic* = 2.666, *P* = 0.005) and by 18 ± 4% in 14 slices from four aged mice (*Z-Statistic* = 3.233, *P* = 0.002, Figures 4C,D). There was a significant difference in the effect of quinpirole on γ power between young and aged mice (*U* = 27.000, *P* = 0.013). These results indicate that D2-like receptor activation enhances γ oscillations in hippocampal slices from both young and aged mice, but to a larger extend in young mice.

### Selective Dopamine Receptors 3 Activation Does Not Affect γ Oscillations

**FIGURE 5:**
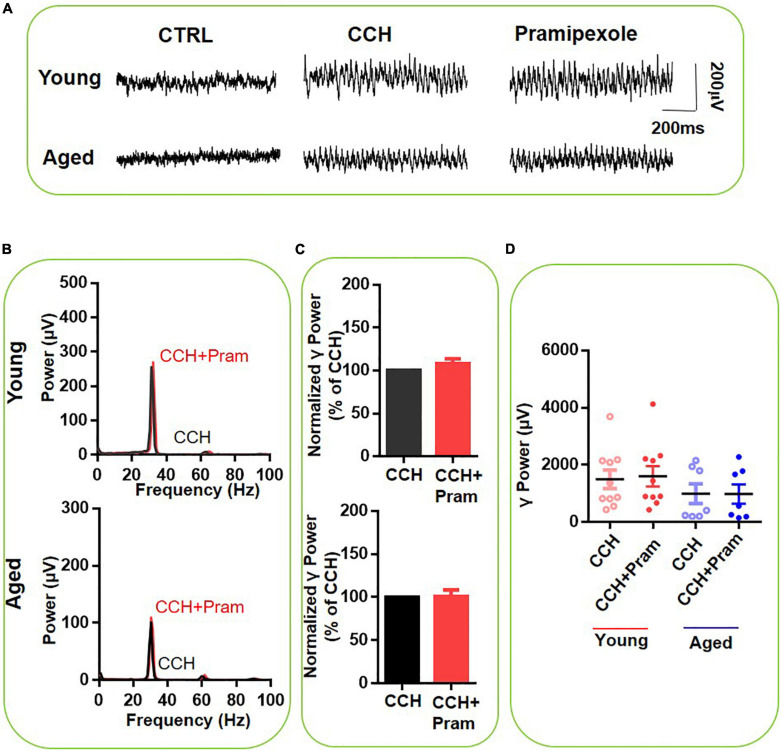
The influence of Pramipexole on CCH-induced γ oscillations in the young and aged mice. **(A)** Example of γ oscillations recorded in area CA3, induced by CCH (5 μM) and after addition of Pramipexole (10 μM). **(B)** Power spectra of the oscillatory activity in CCH alone (black line) and after Pramipexole application (red line) for the slice in **(A)**. **(C)** The γ power normalized to the CCH only baseline value for CCH only application (control) and for Pramipexole application. **(D)** Scatter plots of hippocampal γ power (summated power in 20–60 Hz range) in young and aged mice. The red circles represent CCH-induced γ power before (empty) and after (full) application of Pramipexole from the young mice. The blue circle represents the CCH-induced γ power before (empty) and after (full) application of Pramipexole from the aged mice [vs. CCH control, *t*_(9)_ = –2.123, *P* = 0.063 for young mice; *t*_(6)_ = 0.185, *P* = 0.859 for aged mice]. Data are expressed as mean ± SEM.

We next investigated the contribution of DR3 activation in the DA effect, by testing the effect of the selective DR3 agonist pramipexole (10μM) on CCH-induced γ-oscillations. Figure 5 gives an example of the effect of pramipexole on the γ oscillation strength in a typical slice from a young mouse (top panels) and an aged mouse (bottom panels). The power spectrum of the oscillation in the slice from the young mouse (Figure 5B, top panel) and of that from the aged mouse (Figure 5B, bottom panel) show no change in peak frequency. Pramipexole had no effect on hippocampal γ power of either young mice [108 ± 8% of baseline power, *t*_(9)_ = –2.123, *P* = 0.063] or aged mice [100 ± 9% of baseline power, *t*_(6)_ = 0.185, *P* = 0.859, Figures 5C,D]. These results indicated that DR3 do not contribute to the effect of DA or quinpirole on mouse hippocampal γ oscillations.

### Selective Dopamine 4 Receptor Activation Increases γ Oscillations More in Slices From Aged Mice Than in Slices From Young Mice

**FIGURE 6:**
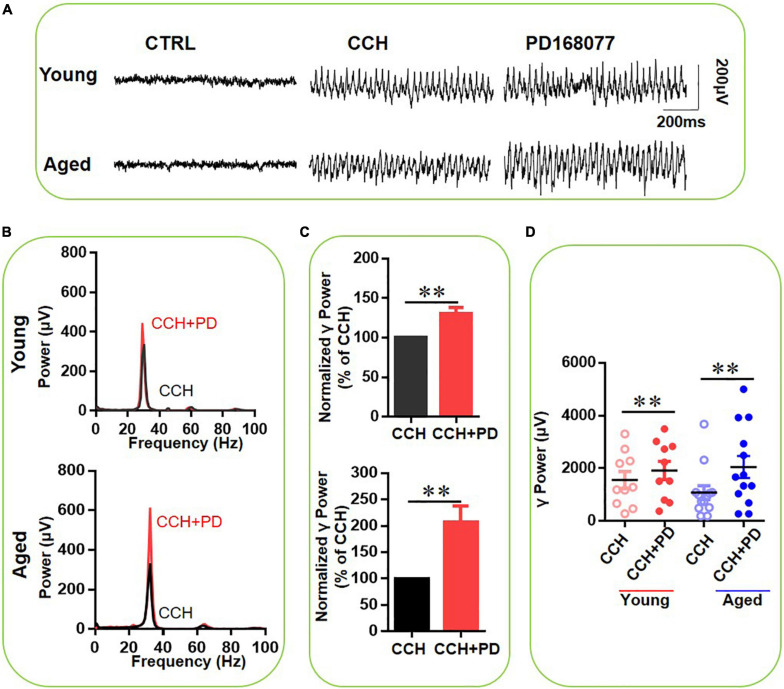
The influence of PD-168077 on CCH-induced γ oscillations in the young and aged mice. **(A)** Example of γ oscillations recorded in area CA3, induced by CCH (5 μM) and after addition of PD-168077 (200 nM). **(B)** Power spectra of the oscillatory activity in CCH alone (black line) and after PD-168077 application (red line) for the slice in **(A)**. **(C)** The γ power normalized to the CCH only baseline value for CCH only application (control) and for PD-168077 application. **(D)** Scatter plots of hippocampal γ power (summated power in 20–60 Hz range) in young and aged mice. The red circles represent CCH-induced γ power before (empty) and after (full) application of PD-168077 from the young mice. The blue circle represents the CCH-induced γ power before (empty) and after (full) application of PD-168077 from the aged mice [vs. CCH control, *t*_(9)_ = –3.799, ^**^P = 0.004 for young mice; *t*_(12)_ = –3.337, ^**^P = 0.006 for aged mice]. Data are expressed as mean ± SEM.

Finally, we assessed the role of DR4 activation in the age-dependent effect of DA on CCH-induced γ oscillations, using the selective DR4 agonist PD-168077 (200 nM) after γ oscillations stabilized. Figure 6A gives an example of the effect of PD-168077 on the γ oscillation strength in a typical slice from a young mouse (top panels) and an aged mouse (bottom panels). The power spectrum of the oscillation in the slice from the young mouse (Figure 6B, top panel) and of that from the aged mouse (Figure 6B, bottom panel) show no change in peak frequency. PD-168077 slightly increased γ power in 10 slices from four young mice by 22 ± 5% from baseline [*t*_(9)_ = –3.799, *P* = 0.004], and dramatically increased γ power in 13 slices from four aged mice by 107 ± 29% [*t*_(12)_ = –3.337, *P* = 0.006, Figures 6C,D]. The γ power increase with DR4 activation was significantly higher in slices from aged mice compared to that in slices from young rats [*t*_(21)_ = –2.452, *P* = 0.023]. These results suggest that the increase in γ oscillations, observed with DA or quinpirole application is in slices from aged mice mainly due to DR4 activation.

**FIGURE 7:**
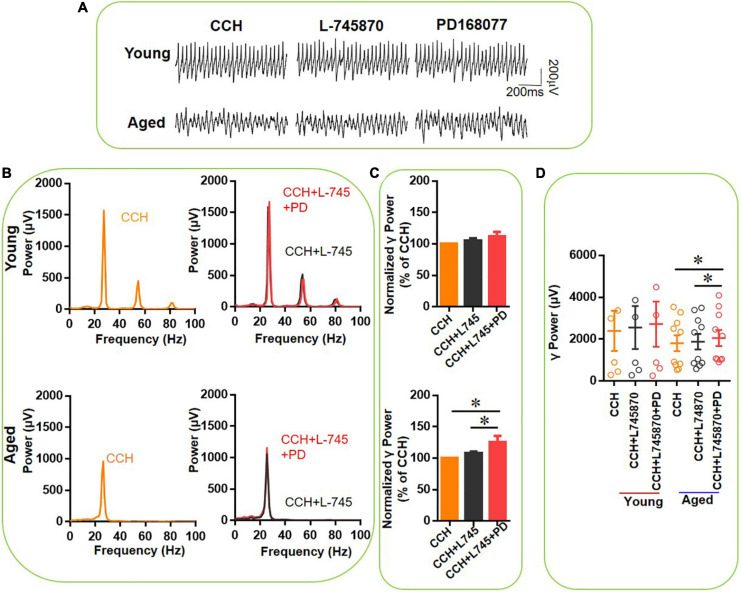
The effect of L-745,870 on PD-168077-mediated increase of CCH-induced γ oscillations in the young and aged mice. **(A)** Example of γ oscillations recorded in area CA3, induced by CCH (5 μM) and L-745,870 (500 nM), and after addition of PD-168077 (200 nM). **(B)** Power spectra of the oscillatory activity in CCH (yellow) and L-745,870 (black line), and after PD-168077 application (red line) for the slice in **(A)**. **(C)** The γ power normalized to the CCH only baseline value for CCH control, CCH + L-745,870 and CCH + L-745,870 + PD-168077 application. **(D)** Scatter plots of hippocampal γ power (summated power in 20–60 Hz range) in young and aged mice. The yellow, black and red empty circles represent γ power values for CCH alone, CCH + L-745,870 and CCH + L-745,870 + PD-168077 application, respectively, from the young and aged mice [vs. CCH + L-745,870, *t*_(5)_ = –1.734, *P* = 0.144, for young mice; *t*_(9)_ = 2.918, *P = 0.025 for aged mice]. Data are expressed as mean ± SEM.

**FIGURE 8:**
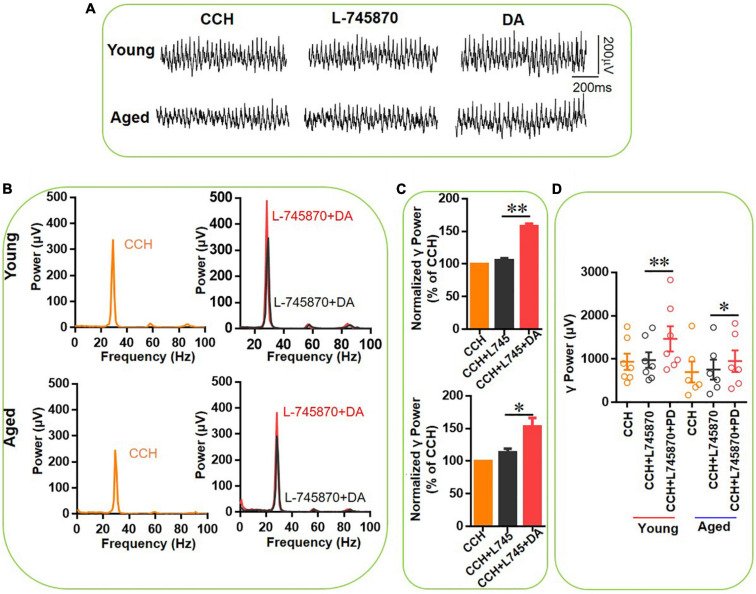
The effect of L-745,870 on DA-mediated increase of CCH-induced γ oscillations in the young and aged mice. **(A)** Example of γ oscillations recorded in area CA3, induced by CCH (5 μM) and L-745,870 (500 nM), and after addition of DA (200 μM). **(B)** Power spectra of the oscillatory activity in CCH (yellow) and L-745,870 (black line), and after DA application (red line) for the slice in **(A)**. **(C)** The γ power normalized to the CCH baseline value for CCH control, CCH + L-745,870 and CCH + L-745,870 + DA application. **(D)** Scatter plots of hippocampal γ power (summated power in 20–60 Hz range) in young and aged mice. The yellow, black and red empty circles represent γ power values for CCH alone, CCH + L-745,870 and CCH + L-745,870 + DA application, respectively, from the young and aged mice [vs. CCH + L-745,870, *t*_(6)_ = –4.155, ^**^P = 0.006, for young mice; *t*_(5)_ = –3.006, *P = 0.030 for aged mice]. Data are expressed as mean ± SEM.

To verify the receptor selectivity of the age-dependent effect of PD-168077, we tested the effect of the selective DR4 antagonist L-745870 (Miyauchi et al., 2017) on the effect of PD-168077 and on the effect of DA. After the CCH-induced γ oscillation was stable, first L-745870 (500 nM) was applied for 20 min, after which PD-168077 (200 nM) was applied for a time period of reaching a steady state for 20 min. L-745870 alone had no effect on γ power in slices from young rats [105 ± 4% of baseline power, *t*_(5)_ = –1.840, *P* = 0.125, Figure 7A top panels] or aged mice [108 ± 3% of baseline power, *t*_(9)_ = 1.158, *P* = 0.262, Figure 7A bottom panels] and did not affect the peak frequency (Figure 7B). In another set of experiments, application of L-745870 had no effect on CCH-induced γ oscillation (Figures 8A,B). In the presence of L-745870, PD-168077 increased the hippocampal γ power in 10 slices from three aged mice by only 17 ± 8% [*t*_(9)_ = 2.918, *P* = 0.025], PD-168077 had no effect on γ power in slices from young mice [112 ± 7% of baseline power, *t*_(5)_ = –1.734, *P* = 0.144, Figures 7C,D] and there was no age-dependent difference in the effect of PD168077 [*t*_(14)_ = –1.005, *P* = 0.332].

In a similar experiment we tested the effect of DA (200 μM) in addition to L-745870. In the presence of L-745870, DA increased the hippocampal γ power in 7 slices from three young mice by only 49 ± 5% [*t*_(6)_ = –4.155, *P* = 0.006, Figures 8C,D] and in 6 slices from three aged mice by 34 ± 9% [*t*_(5)_ = –3.006, *P* = 0.030, Figures 8C,D] and there was no age-dependent difference in the effect of DA [*t*_(11)_ = 0.347, *P* = 0.735]. Compared with the effect of DA alone in γ power for young (128 ± 26%) and aged mice (185 ± 43%), DA-induced increase of γ power in the presence of L-745870 are significant less than that of DA alone for both young (*U* = 62.000, *P* = 0.037) and aged mice (*U* = 56.000, *P* = 0.045).

These results indicate that DR4 activation is responsible for the age-dependent effect of PD-168077 on γ oscillations and that a significant part of the γ increment by DA is mediated by DR4 activation.

### The Effect of NMDAR Antagonist D-AP5 on PD-168077 Modulation of γ Oscillation

**FIGURE 9:**
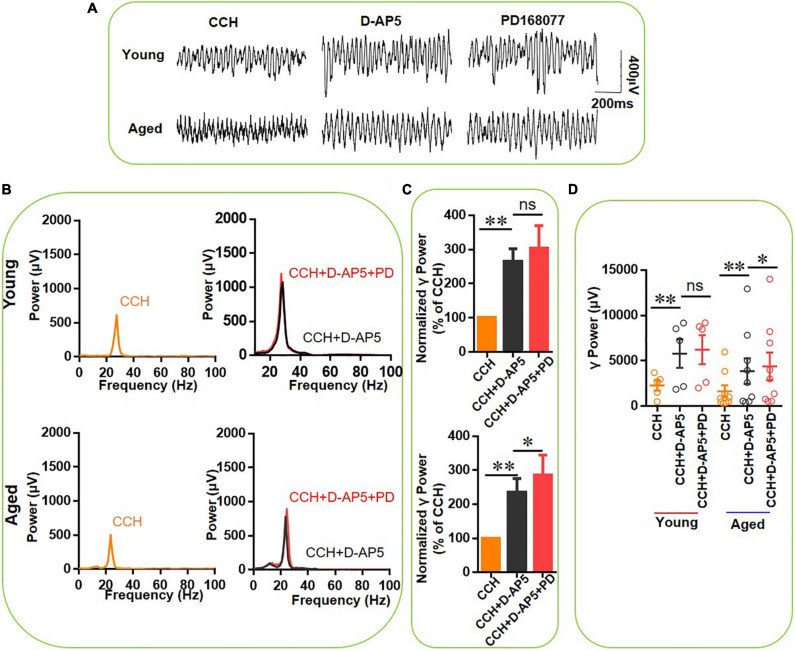
The effect of D-AP5 on PD-168077-mediated increase of increase of CCH-induced γ oscillations in the young and aged mice. **(A)** Example of γ oscillations recorded in area CA3, induced by CCH (5 μM) and D-AP5 (50 μM), and after addition of PD-168077 (200 nM). **(B)** Power spectra of the oscillatory activity in CCH and D-AP5 (black line), and after PD-168077 application (red line) for the slice in **(A)**. **(C)** The γ power normalized to the CCH and D-AP5 baseline value for CCH and D-AP5 application (control) and for PD-168077 application (Young: *P* = 0.320, paired *t*-test, *n* = 8; aged: *P* = 0.079, paired *t*-test, *n* = 11). **(D)** Scatter plots of hippocampal γ power (summated power in 20–60 Hz range) in young and aged mice. The yellow, black and red empty circles represent γ power values for CCH alone, CCH + D-AP5 and CCH + D-AP5 + PD application, respectively, from the young and aged mice [D-AP5 vs. CCH, *t*(4) = 3.914, ^**^P = 0.00446, CCH + D-AP5 + PD vs. D-AP5, *t*_(4)_ = 0.47, *P* = 0.651 for young mice; D-AP5 vs. CCH, *t*(8) = 3.085, ^**^P = 0.0071, CCH + D-AP5 + PD vs. D-AP5, *t*(8) = –2.564, *P = 0.033 for aged mice]. Data are expressed as mean ± SEM.

DR4 stimulation reduces NMDAR transmission (Kotecha et al., 2002) and NMDAR antagonism increases kainate-induced γ oscillation strength and occludes the γ oscillation-enhancing effect of DR activation in slices from young mice (Li et al., 2019) and rats (Andersson et al., 2012a). To test whether NMDAR antagonism can prevent the DR4-mediated enhancement of CCH-induced γ oscillations in slices from aged mice, we applied first the NMDAR blocker D-AP5 (50 μM) for a time period with a steady state reached for 20 min, followed by PD168077 (200 nM). Figure 9A demonstrates the effect of D-AP5 on CCH-induced γ oscillations in a typical slice from a young mouse (top panels) and from an aged mouse (bottom panels) and the lack of effect on the peak frequency (Figure 9B). D-AP5 alone increased hippocampal γ power by 164 ± 38% from baseline [*t*_(4)_ = 3.914, *P* = 0.00446] in five slices from young mice and by 134 ± 42% [*t*_(8)_ = 3.085, *P* = 0.0071] in nine slices from aged mice (Figure 9C).

In the presence of D-AP5, PD-168077 did not change γ power in slices from young mice [110 ± 9% of D-AP5 power, *t*_(4)_ = 0.47, *P* = 0.651, example in Figures 9A,D], which was not different from the effect of PD-168077 alone [122 ± 5%, *t*_(13)_ = –1.216, *P* = 0.246, Figure 9C]. In slices from aged animals, the addition of PD-168077, caused an additional 20 ± 5% increase in γ power [*t*_(8)_ = –2.564, *P* = 0.033, example in Figure 9A], which is significantly less than the effect of PD-168077 alone (*U* = 100, *P* = 0.006). These results indicate that the NMDAR antagonist largely occludes the effect of the DR4 agonist, suggesting that the modulation of γ oscillations by D4Rs requires the inactivation of NMDARs.

## Discussion

In this study, we found that (1) CCh-induced γ oscillations were impaired in aged mice, which can be restored by exogenous application of DA; (2) DR1 activation increased γ power in young mice but had little effect in aged mice, D2-like receptor activation enhanced γ oscillations in both young and aged mice, which was in aged mice mainly due to DR4 activation; (3) the effect of DR4 on γ oscillations was occluded by an NMDAR antagonist.

### Gamma Oscillations Are Reduced With Aging

The age-dependent reduction in γ power of CCH-induced oscillations in mouse hippocampal slices may indicate changes in the γ oscillation-generating neuronal network and/or in the excitability of its mayor neuronal cell types. Carbachol is known to drive pyramidal cell activity through the muscarinic M1 receptor (Fisahn et al., 2002) and the resulting γ oscillation is facilitated by nicotinic receptor activation (Stoiljkovic et al., 2015; Wang et al., 2017). This may be explained in part by an aging-associated reduction in M1 receptor expression (Amenta et al., 1994) and nicotinic receptor expression (Gahring et al., 2005). However, since age-related reductions in γ power were also reported in the kainate model of gamma oscillations (Vreugdenhil and Toescu, 2005), changes in the neuronal network are likely to contribute. Interestingly, gamma power is also reduced in the EEG of aged humans (Murty et al., 2020), validating the *ex vivo* model used in this study, and justifying the speculation that the ageing-related cognitive decline, attributed to cholinergic hypofunction (Terry and Buccafusco, 2003), is in part due to impaired γ synchronization of neuronal activity.

### Dopamine Receptors Differentially Modulate γ Oscillations in Young and Aged Mice

DR1 is a D1-like receptor, mainly expressed on principal cells in the ventral part of the hippocampus (Papaleonidopoulos et al., 2018). DR1 activation stimulates adenylate cyclase and PKA activity, which would increase principal cell excitability driving CCH-induced γ oscillations (Fisahn et al., 2002). In addition, the DR1 activation-induced receptor tyrosine kinase-ERK pathway has been implicated in enhancing γ oscillations in rat hippocampal slices (Xie et al., 2021). DR1 activation has little effect on γ oscillations in slices from aged mice, which can partly be explained by aging-related reduction of DR1 expression observed in rodents (Guo et al., 2017; Zhang et al., 2021) and humans (Karrer et al., 2017), but may also involve changes in post-receptor signaling pathways.

In contrast to D1-like receptor expression, D2-like receptor expression does not decrease with aging in human hippocampus (Seaman et al., 2019) and rodent area CA3 (Amenta et al., 2001). D2-like dopamine receptors are mainly expressed on interneurons in the cortex (Cousineau et al., 2020) and hippocampus (Andersson et al., 2012b) D2-like receptors inhibit adenylate cyclase activity and although this could reduce excitability, D2-like receptor activation increases fast-spiking interneuron activity and GABAergic inhibition (Tseng and O’Donnell, 2007; Tomasella et al., 2018), probably through a β-Arrestin2–dependent inhibition of Akt and activation of Gsk3β (Urs et al., 2016). In Addition, D2-like receptors can activate receptor tyrosine kinase-ERK signaling (Kim et al., 2006; Beaulieu et al., 2011), which is critical for dopamine-induced enhancement of γ oscillation in young rats (Xie et al., 2021). Quinpirole has high affinity for DR2 as well as for DR3 and DR4 (Surmeier et al., 1996). Since the quinpirole-mediated increase in γ oscillations was not different between young and aged mice, the selective DR3 activation had no effect and the selective DR4-mediated increase in γ oscillations was stronger in aged mice, the quinpirole-mediated increase is likely to be mediated mainly by DR2 in young mice and mainly by DR4 in aged mice.

### Dopamine 4 Receptor Activation Enhances γ Oscillations in Aged Mice Through an NMDAR Dependent Mechanism

DR4 is the most abundant D2-like DR in the hippocampus and DR4 is prominently expressed in PV-interneurons (Andersson et al., 2012b). DR4 activation increases γ oscillations in rat hippocampus (Andersson et al., 2012b) and primate prefrontal cortex (Nakazawa et al., 2015). This has been attributed to DA simultaneously reducing spontaneous firing and increasing evoked firing in PV-expressing interneurons (Tierney et al., 2008; Furth et al., 2013), which increases temporal precision and synchronization of fast-spiking interneuron firing (Andersson et al., 2012a), crucial in γ oscillations. Interestingly, sepsis causes reduced γ oscillation and consequent cognitive impairments *via* a reduction in DR4 and PV-expressing interneuron activity (Ji et al., 2020). The signaling pathway downstream of DR4 activation is complex and involves synergistic action with the Neuregulin/ErbB4 signaling (Andersson et al., 2012b). DR4 activation causes inhibition of NMDAR transmission through NMDAR downregulation *via* transactivation of a receptor tyrosine kinase and downstream activation of phospholipase C/inositol triphosphate/Ca^2+^ signaling (Kotecha et al., 2002). Inhibition of NMDAR transmission by NMDAR antagonists increases kainate-induced γ oscillation strength (Mann et al., 2005; Li et al., 2019) and *in vivo*γ oscillations (Kealy et al., 2017). NMDAR antagonism occludes the γ oscillation-enhancing effect of DR activation by methamphetamine in slices from young mice and rats (Li et al., 2019). Interestingly, D-AP5 not only boosted γ oscillations, especially in slices from aged mice, but largely prevented further enhancement by DR4 activation, similar to what was reported in slices from adult rats (Andersson et al., 2012a). This occluding effect of NMDAR antagonism suggests that the DR4-mediated enhancement of γ oscillations, involves NMDAR inactivation. Since hippocampal expression of DR4 does not change with aging (Guo et al., 2017), it is surprising that the DR4-mediated enhancement of γ oscillations is so much stronger in slices from aged mice than in those from young mice. It points to age-dependent changes in the DR4—NMDAR signaling pathway, which needs further investigation.

### Implications of This Study

The age-dependent impairment of hippocampal γ oscillations that is associated with aging-related cognitive decline, can be restored by exogenous application of DA. Interestingly, stimulating DRs with amphetamine improves cognitive functions in the elderly more than the young (Garrett et al., 2015). Our study suggests that selective DR4 agonists may be even more beneficial in counteracting age-related cognitive decline.

The age-related difference in the regulation of γ oscillations by different DR subtypes provides new insight for the understanding of changed DR function in normal aging. Elucidating the post-DR4 signaling pathways that can potentiate the γ oscillation-enhancing effect of DA, may identify potential targets for cognition-boosting therapies in normal aging and aging-related diseases like Alzheimer’s disease.

## Data Availability Statement

The original contributions presented in the study are included in the article/supplementary material, further inquiries can be directed to the corresponding author/s.

## Ethics Statement

The animal study was reviewed and approved by the Ethics Committees of Xinxiang Medical University.

## Author Contributions

Y-KJ, YW, Z-RL, and B-YF conducted the experiments. Y-KJ, YW, T-CG, Z-RL, B-YF, and J-HH analyzed the data. MV supervised the experiments, analyzed the data, and revised the manuscript. C-BL designed the experiments, analyzed the data, wrote, and revised the manuscript. All authors contributed to the article and approved the submitted version.

## Conflict of Interest

The authors declare that the research was conducted in the absence of any commercial or financial relationships that could be construed as a potential conflict of interest.

## Publisher’s Note

All claims expressed in this article are solely those of the authors and do not necessarily represent those of their affiliated organizations, or those of the publisher, the editors and the reviewers. Any product that may be evaluated in this article, or claim that may be made by its manufacturer, is not guaranteed or endorsed by the publisher.
